# A learning mechanism shaping risk preferences and a preliminary test of its relationship with psychopathic traits

**DOI:** 10.1038/s41598-021-00358-8

**Published:** 2021-10-21

**Authors:** Takeyuki Oba, Kentaro Katahira, Hideki Ohira

**Affiliations:** 1 Department of Psychology, Graduate School of Environmental Studies, Nagoya University, Furo-cho, Chikusa-ku, Nagoya, 464-8601 Japan; 2 Department of Psychology, Graduate School of Informatics, Nagoya University, Furo-cho, Chikusa-ku, Nagoya, 464-8601 Japan

**Keywords:** Decision, Personality, Learning algorithms

## Abstract

People tend to avoid risk in the domain of gains but take risks in the domain of losses; this is called the reflection effect. Formal theories of decision-making have provided important perspectives on risk preferences, but how individuals acquire risk preferences through experiences remains unknown. In the present study, we used reinforcement learning (RL) models to examine the learning processes that can shape attitudes toward risk in both domains. In addition, relationships between learning parameters and personality traits were investigated. Fifty-one participants performed a learning task, and we examined learning parameters and risk preference in each domain. Our results revealed that an RL model that included a nonlinear subjective utility parameter and differential learning rates for positive and negative prediction errors exhibited better fit than other models and that these parameters independently predicted risk preferences and the reflection effect. Regarding personality traits, although the sample sizes may be too small to test personality traits, increased primary psychopathy scores could be linked with decreased learning rates for positive prediction error in loss conditions among participants who had low anxiety traits. The present findings not only contribute to understanding how decision-making in risky conditions is influenced by past experiences but also provide insights into certain psychiatric problems.

## Introduction

Decision-making under uncertainty is ubiquitous; individuals often choose between an option with low variance but a smaller outcome (i.e., a sure option) and an option with high variance but a larger outcome (i.e., a risky option). In situations where possible outcomes and their probabilities are explicitly given (i.e., a descriptive choice), people generally tend to avoid risky options in the domain of gains but take the risky option in the domain of losses^1,2^. This preference pattern is called the reflection effect, and prospect theory, which is the most influential theory for decision-making under risks, uses nonlinear subjective value to account for it^1^.

In contrast, learning processes that can shape attitudes toward risk through experience (i.e., an experience-based choice) in both domains have been proposed. Many studies have examined experience-based decisions because it is not always the case that a decision-maker has access to all the relevant information, such as the results and their probabilities. The results from these studies have revealed that risk preference under experience-based choice is different from that under descriptive-based choice. For example, in the choice between the option of sure $3 gain and the risky option including $4 gain with an 80% probability, people tend to select the sure option in the descriptive condition, but they tend to choose the risk option in the experience condition^2,3^. This discrepancy is called the description-experience gap^4^ and is often thought of as a difference in the distortion of subjective probabilities; that is, rare events are more likely to influence decisions in the description-based choice than their objective probabilities and less likely to influence decisions in the experience-based choice. A recent meta-analytic review has shown that the description-experience gap is robust, especially when (1) the decisions include one risky option and one sure option, (2) the decisions are associated with the loss domain or mixed gains and losses, and (3) the probability of rare events is very low and that there are several possible causes, such as a sampling error in estimating probability distribution and a recency effect of outcomes^4^. However, the dynamic processes underlying how individuals learn the value of risky options that lead the risk preference and the difference in those processes between the gain and loss domains are still unclear. It is important to understand how risk preference is formed through experience because a change in risk preference seems to be related to socioeconomic and psychiatric problems^5,6^.

Recent research has used reinforcement learning (RL) models to investigate the influences of risk on the learning processes underlying decision-making. A key component in RL models is prediction error (PE), which is the difference between anticipated and observed values^7^. PE is positive when the received values exceed predictions, whereas PE is negative when the received values fall below predictions. Although several theories proposed to explain risk preference have assumed that the amount of change in the subjective value of outcomes is nonlinear^1,8^, Niv, Edlund, Dayan & O’Doherty^9^ reported that in a reward-based learning task, an RL model explained risk sensitivities by the contrast between the magnitude of learning rates for positive and negative PEs rather than by nonlinear subjective value. This finding indicated that in the domain of gains, the risk preference is a function of the ratio of learning rates for positive and negative PEs. Niv et al.^9^ tested RL models that contained either the learning rates for signed PEs or the subjective utility parameter; however, they did not examine whether these two sets of learning parameters can together influence risk sensitivities.

As in the result of Niv et al.^9^, the magnitude of the learning rate is often different for positive PE and negative PE. Gershman^10^, for example, examined whether the learning rate varied with reward probability and showed that for any given reward probability, the learning rate for negative PE was greater than that for positive PE. The separation in learning rates between positive and negative PEs can represent the extent to which an individual learns when a recent outcome was better than expected (i.e., positive PE) or worse than expected (i.e., negative PE). In one simulation study, a strong bias in the learning rate for positive PE improves learning performance in situations where the reward probabilities are very low but decreases learning performance in situations where the reward probabilities are very high^11^. These relationships are reversed when the learning rate for negative PE is larger than that for positive PE. The separate learning rates can tell us the bias of the learning process.

In contrast to the domain of gain, it is still unknown whether learning mechanisms that shape risk preference in the domain of losses derive from nonlinear subjective values, asymmetric effects of learning rates for positive and negative PEs, or both. Several studies of decision-making in loss conditions have assumed nonlinear subjective values^1,5^, and risk-seeking behavior was interpreted as a sharp decline in sensitivity to increased loss. Thus, the nonlinearity of subjective values in the domain of losses may explain the learning of a risk preference. In contrast, if Niv et al.'s results can apply to a learning task in the domain of losses, learning rates can also play important roles. For example, persons who have a higher learning rate for positive P E t han for negative PE tend to choose risky options through learning because of updating processes in which the persons emphasize the experience of the good side of risky options (e.g., omission of a punishment) more than the bad side of risky options (e.g., a greater amount of a loss). Therefore, it can also be hypothesized that risky choices under loss might stem from a higher learning rate for positive PE than for negative PE.

Understanding the learning mechanisms underlying risk preference under loss can shed new light on understanding the mechanisms of certain psychopathologies, specifically psychopathy. Psychopathy is a group of personality traits constituted by maladaptive interpersonal and affective features and poor behavioral inhibition^12^, and affective features are frequently regarded as the central characteristics of psychopathy. Previous studies have revealed that individuals with psychopathy exhibited poor performance in the punishment-based learning task, especially when they had lower anxiety traits^13,14^. Regarding the learning processes, Oba, Katahira and Ohira^15^ showed that individuals with high psychopathic traits have lower learning rates for positive PE in a loss condition compared to those with low psychopathic traits, indicating that individuals with psychopathic traits were less likely to learn from avoidance of a negative outcome. However, they did not test whether psychopathic traits were related to the nonlinear subjective value for losses. Newman, MacCoon, Vaughn, & Sadeh^16^ reported that compared to a control group, psychopathic individuals had lower sensitivity to punishment when they had low anxiety. This suggests that individuals with high psychopathy but low anxiety traits may show lower sensitivity to changes in losses. We investigated the relationships between psychopathic traits and learning parameters, including nonlinear subjective values and learning rates.

In summary, the current study aims to investigate the learning mechanisms that shape risk preference in gain and loss domains. In addition, we examined the relationship between these learning parameters and psychopathy-related personality traits. With respect to acquiring risk preferences, we hypothesized that based on the results of experience-based choice studies^2,3^, under conditions of choosing between sure and risky options that have the same expected value, the risky option would often be chosen in the domain of gains but would not frequently be selected in the domain of losses (i.e., the reflection effect). The conventional theories in decision-making predict that risk preference is related to nonlinear subjective values. In contrast, according to Niv et al.^9^, the contrast in learning rates for positive and negative PEs can also predict risky decision-making in a learning task. It is possible to consider that a greater learning rate for positive PE than for negative PE contributes to risk seeking, while a greater learning rate for negative PE than for positive PE leads to risk aversion. If the reflection effect is observed, a learning rate for positive PE in the gain domain may be larger than that for negative PE, while a learning rate for positive PE could be smaller than that for negative PE in the domain of losses. Moreover, we examined a hybrid model that combined the effects of both learning rates and nonlinear subjective values. We performed a model comparison to confirm which model was the best fitting and investigated the above hypotheses. Regarding personality traits, the learning characteristics of individuals with psychopathy can be modified by anxiety; that is, in the loss domain, psychopathic traits are negatively related to the learning rate for positive PE and the subjective utility parameter when individuals have low anxiety traits. However, we note that the sample size in this study may be too small (N = 51) to test the relationship between personality traits and learning parameters. Therefore the results related to personality traits should be regarded as preliminary.

## Results

**Figure 1:**
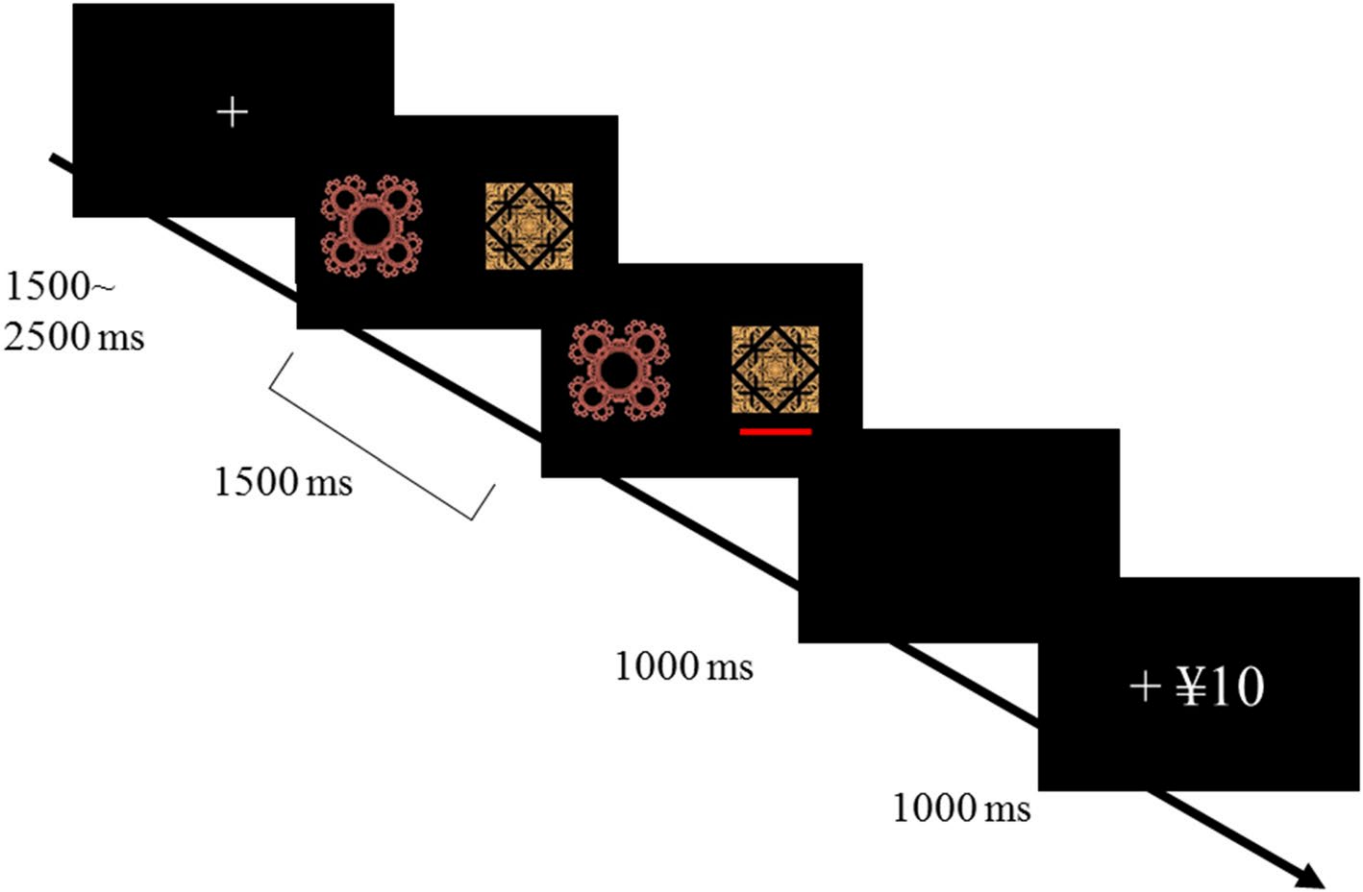
Learning task used in this experiment. A sample of a gain trial is depicted.

Fifty-one participants performed a learning task and completed questionnaires that investigated personality traits. We briefly explain the learning task and questionnaires. In the learning task, the participants were required to choose one of the two options that were associated with different monetary outcomes (Fig. 1; see Methods for details). The learning task was divided into gain and loss blocks. In each block, there were 5 options, including two sure ¥0 options, one sure ¥10 option, one sure ¥20 option, and one risk option that had ¥0 or ¥20 with a 50% probability, and each combination of the two options was presented 20 times except the combination of the risk and sure ¥10 options that appeared 30 times to test risk preference. The number of trials was set as 210 in each block; thus, there were 420 trials in this experiment. The participants won these amounts of money in the gain block but lost in the loss block. For the questionnaires, the Japanese version of the Levenson Self-report Psychopathy Scale (LSRP^17,18^) and the anxiety trait from the State-Trait Anxiety Inventory (STAI^19,20^) were used to assess the psychopathy and anxiety traits of participants.

### Learning performance

To confirm whether participants had learned to choose an advantageous option, we examined learning performance on trials in which there was a better option. The mean proportion of advantageous responses for each trial type was greater than 0.7 in both the gain and loss domains and significantly higher than the chance level (all *p*s < 0.001 with the Bonferroni correction).

Furthermore, to investigate whether participants chose the better options through learning, the choice data were divided into first half and second half of trials, then we performed a repeated measure analysis of variance (ANOVA) with the 2 (domain: Gain/Loss) × 2 (time point: First half/Second half) × 5 (trial type: ¥0 vs. ¥10/¥0 vs. Risk/¥0 vs. ¥20/¥10 vs. ¥20/Risk vs. ¥20). All the main effects were significant (*F*(1,50) = 8.633, *p* = 0.005, η_p_^2^ = 0.147 for the main effect of domain; *F*(1,50) = 107.021, *p* < 0.001, η_p_^2^ = 0.682 for the main effect of time point; *F*(2.75,137.36 [with the Greenhouse–Geisser correction]) = 40.664, *p* < 0.001, η_p_^2^ = 0.449 for the main effect of trial types). The interaction between the domain and time point was significant (*F*(1,50) = 5.158, *p* = 0.028, η_p_^2^ = 0.094), indicating that in the second half of the learning task, participants made better choices in the gain domain more frequently than in the loss domain (*p* < 0.001). Moreover, in both domains, the advantageous option was more often selected in the second half of the learning task than in the first half of the task (*p*s < 0.001). We also found an interaction effect of domain × trial type (*F*(2.5,125.2 [with the Greenhouse–Geisser correction]) = 18.343, *p* < 0.001, η_p_^2^ = 0.268). This interaction effect showed that in the trials of ¥10 vs. ¥20 and Risk vs. ¥20, the number of choices of advantageous options was greater in the gain domain than in the loss domain (both *p*s < 0.001), while in the ¥0 vs. Risk trial, the proportion of choosing the better option was higher in the loss domain than in the gain domain (*p* < 0.001). In the gain domain, the learning performance in the ¥0 vs. ¥20 trial was greater than that in the other trial types (*p*s < 0.001), and the learning performance in the ¥0 vs. Risk trial was lower than that in the other trials (*p*s < 0.05). In contrast, in the domain of losses, the participants showed a better learning performance in the ¥0 vs. ¥20 trial than in the other types of trials (*p*s < 0.05), and their learning performances in the ¥10 vs. 20 trial and Risk vs. ¥20 trial were worse than in the other trial types (*p* < 0.001). The other interactions did not reach statistical significance (*F*s < 1.9, *p*s > 0.14). These results suggest that participants successfully learned the value of each condition.

Next, we examined the choice between the risky option and the sure ¥10 option. We expected that the number of risky option choices would be greater in the gain domain than in the loss domain, namely, the reflection effect. However, the proportion of the risky option was significantly lower in the gain domain than in the loss domain (Gain: *M* = 0.365, *SD* = 0.233; Loss: *M* = 0.488, *SD* = 0.262; *t*(50) = 2.406, *p* = 0.020, *d* = 0.337). This finding suggested that at least in this study, the reflection effect was consistent with the case of the descriptive choice.

We also tested whether the risk preference changed through learning. A 2 (domain: Gain/Loss) × 2 (time point: First half/Second half) repeated measure ANOVA was conducted for the proportion of risk choice. We found a significant main effect of domain (*F*(1,50) = 5.788, *p* = 0.020, η_p_^2^ = 0.103), indicating that consistent with the above result, the participants chose the risk option in the loss domain more than in the gain domain. The main effect of time was also significant (*F*(1,50) = 7.339, *p* = 0.009, η_p_^2^ = 0.128). This main effect revealed that the number of risk choices decreased from the first half to the second half of the trials. The interaction between domain and time was not significant (*F*(1,50) = 0.009, *p* = 0.926, η_p_^2^ = 0.0002).

### Model comparison

**Figure 2:**
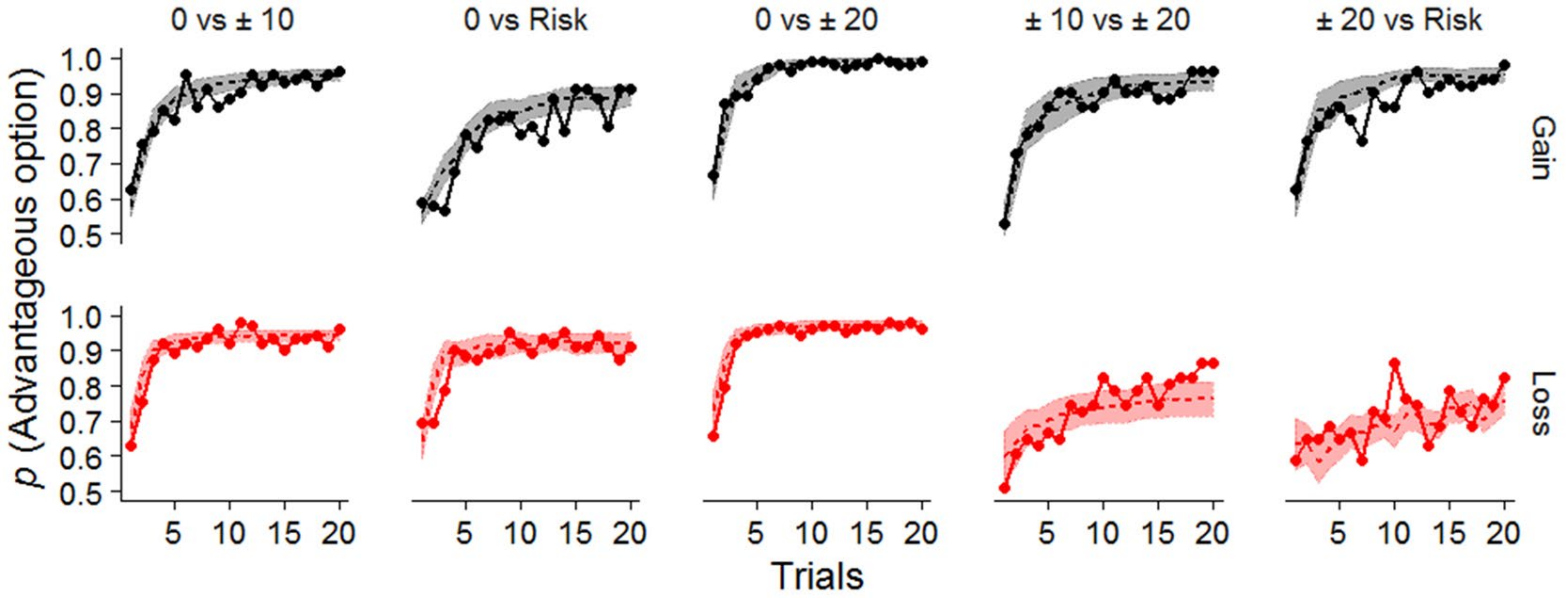
Average probabilities of choosing an advantageous option for each trial type and the model predictions. The solid lines indicate the proportions of participants choosing a better option in each trial across participants, and the dashed lines show the predictions of the winning model. The shaded areas represent the 95% confidence intervals of the model prediction.

We compared the integrated Bayes Information Criteria (iBIC) of each RL model to evaluate the goodness of fit. The iBIC value corresponds to an approximation of the marginal log likelihood computed directly by Monte Carlo sampling (see Methods for details). Table 1 shows the negative log marginal likelihoods and the iBIC values for each model. The hybrid model had a lower iBIC value than the other models even when all learning parameters were separated into the gain domain and loss domain. Figure 2 shows the learning curves and the predictions by the hybrid model for the trials in which there is a better option. The results of the model comparison indicate that participants relied on both the effects of signed PEs and nonlinear subjective values to guide their own choice. In subsequent analyses, we used the learning parameters in the hybrid model that were divided into the gain and loss domains.

**Table 1:** The iBIC values and the negative log marginal likelihoods for the models using all choice data for each participant.

Models	Set of parameters	iBIC (-LML)
Standard model	*α*, *β*	16,868.56 (8414.34)
Separate learning rate model	*α*_P_, *α*_N_, *β*	16,490.25 (8215.21)
Subjective utility model	*α*, *β*, κ	16,570.56 (8255.37)
Hybrid model	*α*_P_, *α*_N_, *β*, κ	16,150.33 (8035.28)
Hybrid model (split β into the domains)	*α*_P_, *α*_N_, *β*_G_, *β*_L_, κ	16,131.21 (8015.74)
Hybrid model (split α into the domains)	*α*_GP_, *α*_GN_, *α*_LP_, *α*_LN_, *β*, κ	15,934.95 (7907.64)
Hybrid model (split κ into the domains)	*α*_P_, *α*_N_, *β*, κ_G_, κ_L_	15,999.22 (7949.75)
Hybrid model (split all parameters into the domains)	*α*_GP_, *α*_GN_, *α*_LP_, *α*_LN_, *β*_G_, *β*_L_, κ_G_, κ_L_	15,697.01 (7768.73)

*Note* *α* = learning rate, *β = *inverse temperature, κ = subjective utility parameter, -LML = negative log marginal likelihood. The subscripts represent the following: P = positive PE, N = negative PE, G = gain domain, L = loss domain.

### Model recovery and parameter recovery

We also performed model recovery and parameter recovery analysis to test whether each learning model can be identified by the true model and whether recovered parameters can be biased by other parameters^21^. For each model, we simulated 100 data points and calculated the iBIC values. The RL parameters used in the simulations were randomly generated by the population-level normal distribution for each parameter that was estimated through the model fitting procedure (see Methods for details). The confusion matrix in Supplementary Fig. S1 demonstrates the values based on the iBIC, and a fitting model was best under a generated model. The results of the matrix revealed that the true model was well fitted.

Next, we tested parameter recovery for the hybrid model (Supplementary Fig. S2). All the correlations between true parameters and recovered parameters seemed to be good (*r*s > 0.550). We also confirmed the correlations between the recovered parameters (Supplementary Tables S1). The largest correlation coefficient in the gain domain was − 0.243 for the relationships between the learning rate for positive PE and the inverse temperature (*p* = 0.015). In contrast, in the loss domain, the largest correlation coefficient was 0.248 for the relationship between learning rate for positive PE and the subjective utility parameter (*p* = 0.013). These correlations suggested the possibility of weak trade-offs between recovered parameters. Nevertheless, the findings of model recovery and parameter recovery indicate that it can be dissociated between a learning process of learning rate and that of subjective utility parameter.

### Relationships between learning parameters and risky decisions

We first checked that the inverse temperature value was large enough because too low inverse temperature parameters led to random choice and may suggest that the participant's decision was not followed by the prediction of RL models. To do this, the hybrid model with the inverse temperature that included the parameter space to negative values was fitted to the data. To examine whether the inverse temperature was negative, we conducted one sample t test on the inverse temperature parameter for each domain and found that the inverse temperature was greater than 0 in both domains (Gain: *M* = 3.512, *SD* = 1.196, *t*(50) = 20.975, *p* < 0.001; Loss: *M* = 3.115, *SD* = 0.876, *t*(50) = 25.403, *p* < 0.001), indicating that the participant made decisions based on the learned value.

**Figure 3:**
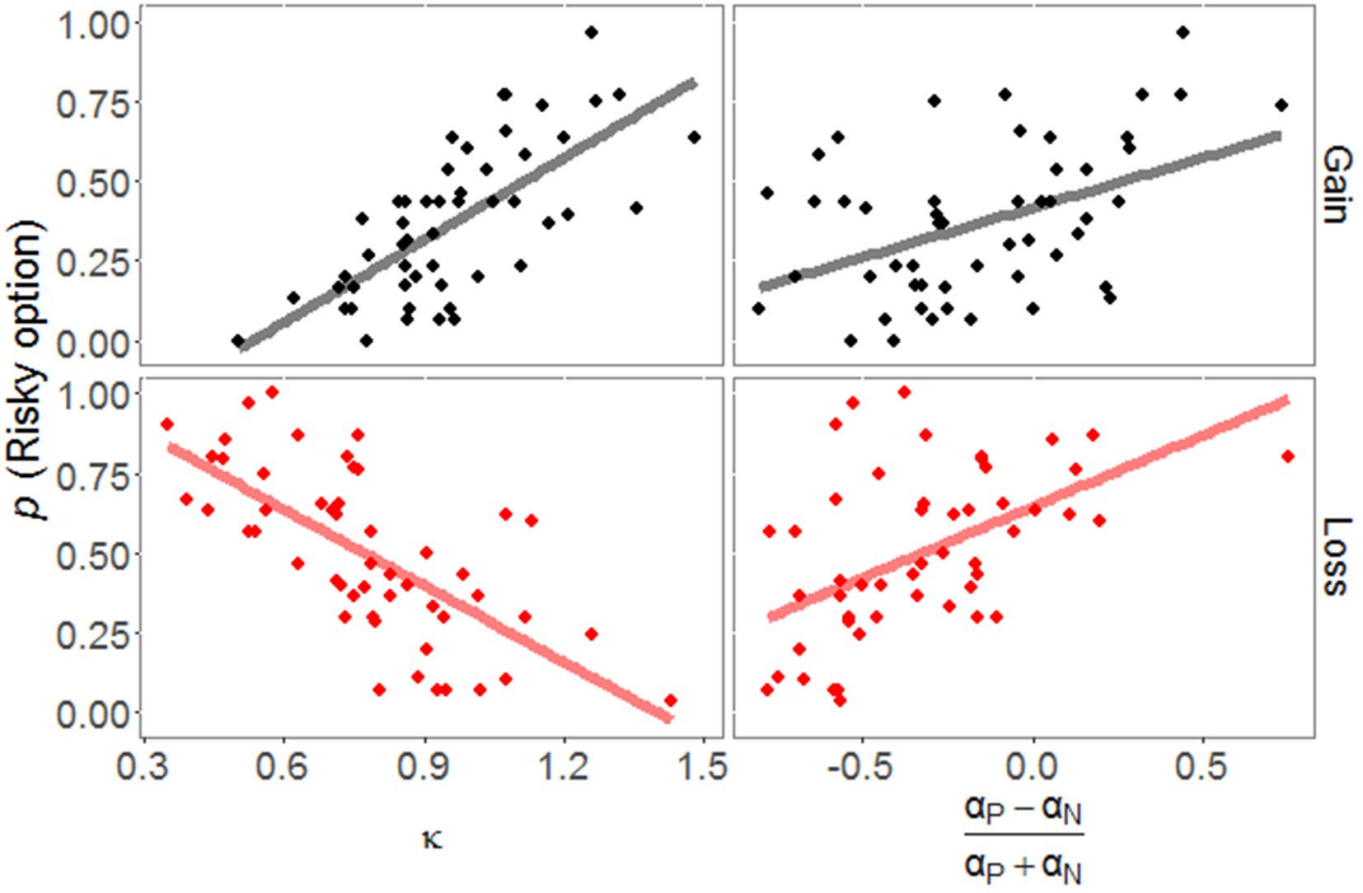
Correlations of the proportion of choosing the risky option with the contrast between learning rates for signed PEs and the subjective utility parameters. The left panels indicate the correlations with the subjective utility parameters, while the right panels show the correlations with the contrast between learning rates for positive and negative PEs.

Following Niv et al.^9^, we calculated a contrast between learning rates for positive and negative PEs ([*α*_p_ − *α*_n_]/[*α*_p_ + *α*_n_]) that represented which of the two learning rates was larger. The contrasts in both domains were correlated with the proportion of risky choices in the condition between the risky option and the sure ¥10 option (Gain: *r* = 0.478, *p* < 0.001, Loss: *r* = 0.517, *p* < 0.001; Fig. 3). These results suggested that the risky option tended to be accepted when the learning rate for positive PE was larger than that for negative PE. The subjective utility parameter for a large outcome was also significantly correlated with the number of choices of the risky option in each domain (Gain: *r* = 0.688, *p* < 0.001, Loss: *r* = -0.681, *p* < 0.001; Fig. 3), indicating that participants who overestimated the large outcome were likely to take the risky option in the domain of gains but to withhold choosing the risky option in the domain of losses. We performed multiple regression analyses to examine whether these parameters were independently related to risk preference. These analyses revealed that both indices were significant in each domain (Gain: *R*^2^ = 0.660, *F*(2,48) = 46.659, *p* < 0.001, *β* = 0.659, *p* < 0.001 for the subjective utility parameter, *β* = 0.433, *p* < 0.001 for the contrast of learning rates; Loss: *R*^2^ = 0.665, *F*(2,48) = 45.640, *p* < 0.001, *β* = − 0.627, *p* < 0.001 for the subjective utility parameter, *β* = 0.441, *p* < 0.001 for the contrast of learning rates).

We tested a priori hypothesis that if the reflection effect was observed, the relationship between the learning rates for positive and negative PEs in the gain domain would be opposite to that in the loss domain. We compared the iBIC values when the hybrid model was not divided into two learning rate parameters for positive PE and negative PE in each domain. If the iBIC value of the hybrid model including the separate learning rates was lower than that of the hybrid model sharing a common learning rate, it suggests the evidence that the learning rates for positive PE and negative PE had the different prior distributions, then, at least the present study, the choice data were predicted by the separate learning rates well. Compared to the iBIC value of the hybrid model with two separate learning rates for the signed PEs in each domain, the iBIC value of the hybrid model with one learning rate in the domain of gains and that of the model with one learning rate in the domain of losses were larger (iBIC: 15,697.01, the negative log marginal likelihood (-LML): 7768.73 for the hybrid model with separate learning rates; iBIC: 15,771.33, -LML: 7815.86 for the hybrid model with one learning rate in the gain domain; iBIC: 15,960.50, -LML: 7910.45 for the hybrid model with one learning rate in the loss domain), indicating that the learning rates were likely to differ between the signed PEs.

We further examined whether the reflection effect was related to the learning parameters. The reflection effect was defined here by subtracting the number of risky choices over the sure ¥10 option in the domain of gains from the number of risky choices over the sure ¥10 option in the domain of losses. The RL parameters that correlated with the risk preference in the loss domain were also subtracted from those in the gain domain. Then, we confirmed the correlation between the reflection effect and these measurements. The difference between the contrasts of learning rates was significantly correlated with the reflection effect (*r* = 0.518, *p* < 0.001), but the difference between the subjective values of large outcomes did not (*r* = − 0.029, *p* = 0.839). However, each subjective utility parameter was negatively correlated with the reflection effect (Gain: *r* = − 0.530, *p* < 0.001; Loss: *r* = − 0.492, *p* < 0.001), indicating that decreasing subjective values for the large outcomes in both domains were related to the extent of the reflection effect.

### Relationships between learning parameters and personality traits

Table 2 shows the correlations between personality traits and learning parameters. In the gain domain, the trait anxiety score was significantly correlated with the inverse temperature parameter (*r* = 0.282, *p* = 0.045), suggesting that the participants with higher anxiety tended to show a lower frequency of random choices. In the domain of losses, secondary psychopathic traits were positively associated with the learning rate for positive PE (*r* = 0.277, *p* = 0.049), indicating that persons who had high secondary psychopathy scores were sensitive to the omission of losses in the risky choice trials. However, these correlations were no longer significant after applying the multiple testing correction.

**Table 2:** The correlations between the learning parameters and the personality traits.

	Descriptive statistics of the personality traits	Gain				Loss			
		*α*_P_	*α*_N_	*β*	κ	*α*_P_	*α*_N_	*β*	κ
PP	*M* = 34.37, *SD* = 5.44, Range[22–45]	− 0.144	0.134	0.122	− 0.033	0.012	0.043	0.069	0.073
SP	*M* = 20.98, *SD* = 3.65, Range[14–29]	− 0.016	0.043	− 0.016	− 0.013	0.277*	− 0.013	− 0.062	− 0.026
TA	*M* = 49.08, *SD* = 11.07, Range[30–70]	0.012	0.228	0.282*	− 0.136	0.107	0.009	0.114	0.061

*Note* PP = primary psychopathy, SP = secondary psychopathy, TA = trait anxiety, *α*_P_ = learning rate for positive PE, *α*_N_ = learning rate for negative PE, *β* = inverse temperature, κ = subjective utility parameter. *p < 0.05.

**Figure 4:**
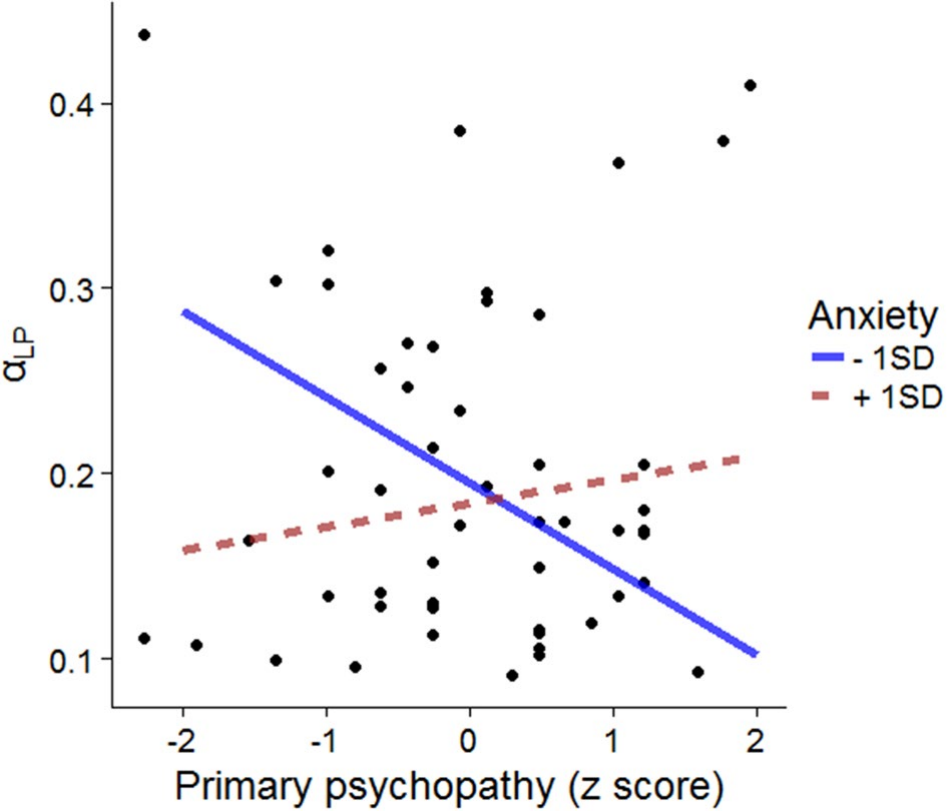
The relationship between the learning rate for positive PE in the domain of losses (*α*_LP_) and primary psychopathy scores interacted with anxiety traits.

We performed hierarchical regression analyses to examine whether these personality traits interacted with each other. After centering these personality trait variables, each factor was included at step 1, and their two-way interactions were entered at step 2. Of interest, the interaction between primary psychopathy and trait anxiety scores was marginally significant on the learning rate for positive PE in the domain of losses (Δ*R*^2^ = 0.187, *F*(3,44) = 3.839, *p* = 0.016, *β* = 0.337, *p* = 0.054; Table 3). We prepared two variables in which the trait anxiety scores 1 SD above and 1 SD below the average were subtracted from individual trait anxiety scores and conducted a simple slope test showing that the learning rate for positive PE in the domain of losses decreased with increased primary psychopathic scores when participants had low anxiety (*β* = − 0.506, *p* = 0.031; Fig. 4) but not high anxiety (*β* = 0.139, *p* = 0.529). Although the interaction effect between primary psychopathy and trait anxiety was not observed under the multiple test correction, if we concentrated on this interaction effect only (that is, the other two interaction terms that did not predict the variance of the learning rate for positive PE in the loss domain were excluded), this interaction effect was still significant even when the multiple test correction was used (*β* = 0.428, *p* = 0.007 with the Bonferroni correction). However, the sample size was too small to conclude the relationship between psychopathy and learning parameters, and this problem will be discussed in a later section.

**Table 3:** Results of hierarchical regression analyses.

		Step 1				Step 2						Δ*R*^2^
		PP	SP	TA	*R*^2^	PP	SP	TA	PP × SP	PP × TA	SP × TA	
		Standardized coefficients				Standardized coefficients						
Gain	*α*_P_	− 0.175	0.069	− 0.013	0.024	− 0.222	− 0.008	0.019	− 0.048	0.129	0.196	0.063
	*α*_N_	0.212	− 0.210	0.321^†^	0.092	0.196	− 0.230	0.385*	− 0.238	0.004	− 0.095	0.071
	*β*	0.256	− 0.346^†^	0.440**	0.161*	0.234	− 0.394*	0.490**	− 0.154	0.099	− 0.014	0.015
	κ	− 0.074	0.112	− 0.188	0.027	− 0.104	0.156	− 0.225	0.112	− 0.302	0.293^†^	0.083
Loss	*α*_P_	− 0.157	0.384*	− 0.074	0.097	− 0.183	0.279	− 0.061	0.052	0.337^†^	0.136	0.187*
	*α*_N_	0.069	− 0.062	0.036	0.004	0.103	0.015	− 0.019	0.143	− 0.176	− 0.046	0.027
	*β*	0.170	− 0.251	0.229	0.054	0.129	− 0.376^†^	0.354*	− 0.379*	0.329^†^	− 0.115	0.108
	κ	0.133	− 0.149	0.128	0.021	0.086	− 0.208	0.214	− 0.283	0.037	0.031	0.060

*Note* PP = primary psychopathy, SP = secondary psychopathy, TA = trait anxiety, *α*_P_ = learning rate for positive PE, *α*_N_ = learning rate for negative PE, *β* = inverse temperature, κ = subjective utility parameter. †*p* < 0.10, *p < 0.05, **p < 0.01.

## Discussion

This study aimed to examine the learning processes of acquiring risk preferences under conditions of gain and loss and to investigate relationships between RL parameters and psychopathic traits. Our data revealed that participants were likely to learn the values of risky options from the effect of different PEs based on nonlinear subjective values. The contrast between learning rates for positive and negative PEs and the subjective utility parameter independently predicted the proportion of risky choices in each domain. We found that in the choice between a risky option and a sure ¥10 option, participants more often chose the risky option in the domain of losses than in the domain of gains (i.e., the reflection effect), and both the learning rates and the subjective utility parameter can also account for the extent to which participants selected risk under losses compared with under gains. Regarding the personality traits, the primary psychopathy scores could interact with the anxiety traits on the learning rate for positive PE in the domain of losses.

The current findings suggested that the effect of signed PEs and the nonlinearity of subjective values was associated with decision-making in risky conditions through experiences. The hybrid model that had separate learning rates for signed PEs and the subjective utility parameter showed a better fit to the choice data than the other models. The proportions of choosing the risky options were independently related to the subjective utility parameter and the contrast between learning rates for positive and negative PEs. These relationships were found in the gain and loss domains. Furthermore, the results of model recovery suggest that the learning process based on separate learning rates can be dissociated from that based on subjective utility. The present findings indicated that risk aversion had formed when the learners had lower sensitivity to the incremental changes in gains and higher sensitivity to increases in loss and/or when they focused more on a negative result of the risky option than on a positive result of the risky option. In contrast, if learners who showed high reward sensitivity and low punishment sensitivity placed more weight on the positive side of the risky choice than on the negative side, they may start to make more risky choices. Our findings were consistent with the results of Niv et al.^9^ and the predictions of decision-making theories^1^, and learning rate and subjective utility parameters appeared to be important for the formation of a risk preference.

Consistent with the findings of Niv et al.^9^ and the results from the descriptive decision^1,2^, participants tended to choose the risky option under the loss conditions more than under the gain conditions (i.e., the reflection effect). In contrast, several studies found that the risk preference in experience-based decisions was opposite that in descriptive decisions^2,3^. This discrepancy tends to increase when the probability of a rare event is low and, in turn, may lead to a sampling error^4^. The probability of the risky option in the current study was relatively high, and the participants learned the value of the risk, at least to the extent that they chose a better option in the choices between a risk and an advantageous or disadvantageous option. Furthermore, the task structure also affects the degree of this difference; that is, the difference is greater when participants can freely terminate their sampling than when participants are forced to sample an outcome^4^. The learning task in this study imposed the latter condition. Therefore, the risk preference in the present study was comparable to that in the descriptive-based decision, and we observed the reflection effect.

While subjective utility has been assumed to be a key mechanism in descriptive decisions and a likely key in experience-based decisions, it has not been clear what mechanisms the learning rate corresponds with. In RL model frameworks, the learning rates can represent how a past event’s outcome influenced the current choice^22,23^. The learning rates for positive PE and negative PE can reflect the temporal effect of desirable and undesirable outcomes, respectively. Previous research has examined the impacts of time sequence on outcomes, particularly the recency effect, which is the tendency to give greater weight to a recent outcome and is supposed to be one of the causes of the difference in risk preference between descriptive and experience-based decisions^3,4^. Although our findings seem to not differ from those for the descriptive choices, we believe that the learning rate parameter may be related to the recency effect, which may engender the description-experience gap.

The learning parameters can represent the factors of the dynamic processes but also capture individual differences in behavioral characteristics. One recent study showed that in the experience-based choice task, participants could be clearly classified into two groups according to the distribution of their learning rates and that participants who had a low learning rate tended to make a decision influenced by rare past results, while those who had a large learning rate tended to choose the risky option in which the punishment could be brought with a low probability^24^. This previous study raised the idea that the distribution of learning parameters can correspond to important indicators of individual differences. In the present study, both the separate learning rates and the subjective utility parameter were widely distributed and correlated with the risk preference well. The computational approach can provide insight into hidden individual differences.

The effects of primary psychopathy on learning rates could be modulated by anxiety traits. Some studies have revealed that the level of anxiety is related to learning performance in psychopathy^13,14^. In particular, learning deficits in psychopathy have often been observed when individuals with high psychopathic traits had low anxiety. The present findings indicated that one learning characteristic associated with lower anxiety and higher psychopathy might be a low learning rate for positive PE under loss conditions, namely, a diminished learning ability when the learner received no punishment when choosing the risky option. This result was partly consistent with those of a previous study^15^. However, the sample size in this study may be insufficient to discuss the effects of psychopathy, and it is highly likely that the other links between psychopathic traits and learning parameters were not detected in this study. We emphasize that the present results related to psychopathy were preliminary data.

We will discuss several limitations of the present study. First, we examined only the condition in which the probability of an outcome being associated with the risky option was 50%. Although the primary aim of the present study was to investigate learning mechanisms underlying the formation of risk preference, it is still unclear what process underlies the difference between descriptive and experience-based decisions that appears when the probability of a rare event is very low^2–4^. We believe that our findings can provide hints to address this difference, but further research is needed. The second limitation is related to the first limitation: we did not compare the risk preference between the learning task and the descriptive task. Future studies should test whether the learning parameters predict the description-experience gap. Third, the sample size was very small for investigating the relationship between learning parameters and personality traits. The small sample size may reduce the statistical power. The other problem related to the results of psychopathy was that the participants in this study were recruited from a non-clinical population. In addition, there were few participants who had extremely high psychopathic traits. Although the concept of psychopathic traits is not limited to a criminal population, the findings of this study on psychopathy should be treated with caution.

In conclusion, our findings provide empirical evidence that risk preference and the reflection effect are likely to be predicted by learning rates for signed PEs and nonlinear subjective values. The learning rate for positive PE in the domain of loss could be related to the extent of primary psychopathy and trait anxiety. The current data can not only contribute to understanding how decision-making in risky conditions is formed by past experiences but also provide insight into psychiatric problems and learning in the face of losses.

## Methods

### Participants

Fifty-one undergraduate students (27 males and 24 females, mean age = 19.51 years, SD = 1.17) participated in this study. All participants provided informed consent and received ¥1,000 for participation. The primary aim of this study is to examine the learning mechanisms for risk preference. Our sample size had more than 80% statistical power to detect a 0.4 correlation coefficient, which seems to be sufficient to test the relationship between the risk preference and the learning parameters. This study was approved by the ethics committee of Nagoya University and conducted in accordance with the relevant guidelines.

### Measurements

The extent of psychopathy was assessed using the Japanese version of the Levenson Self-report Psychopathy Scale (LSRP^17,18^). The LSRP is a 26-item self-report questionnaire that can measure two subcomponents of psychopathy. Primary psychopathy scales include 16 items that represent personality traits such as callousness and a manipulative attitude toward others. The remaining 10 items are comprised of secondary psychopathy scales characterized by impulsivity and stimulus seeking. Each item is rated on a four-point Likert-type scale.

The trait anxiety scale was drawn from the State-Trait Anxiety Inventory (STAI^19^). We used a Japanese version of the STAI^20^. This scale is a 20-item questionnaire that assesses sensitivity to anxiety in daily life using a four-point Likert-type scale.

### Learning task

Participants performed a learning task in which they chose one of two fractal images to which different outcomes were assigned (Fig. 1). On each trial, a fixation cross was present for variable periods, from 1.5 to 2.5 s, followed by a choice stage for 1.5 s. After a blank screen was displayed for 1 s, feedback on the outcome (either of − ¥20, − ¥10, ¥0, + ¥10, or + ¥20) appeared for 1 s, and then, the next trial began. In the choice stage, the participants pressed the F or J key to choose between the two fractals on the two sides of the screen, after which a red bar was displayed under the chosen fractal image. The participants were told that the place where the fractal appeared was not related to the outcome. When participants failed to respond during presentation of the fractal images, feedback appeared to indicate that the response was too late, and a penalty of ¥20 was imposed. The amount of money earned by each participant during the learning task was added to the participation fee, and the participants took this money. Unlike the experiment in Niv et al.^9^, the current experiment did not include training sessions or forced trials to prevent any choice bias.

The experimental task was separated into gain and loss blocks. The order of the blocks was counterbalanced among the participants. Similar to the learning task of Niv et al.^9^, in each block, there were four sure options (two ¥0, ¥10, and ¥20) and one variable risky option (¥0 or ¥20). The participants won these amounts of money in the gain domain but lost money in the loss domain. The probability of the outcome association with the risky option was set at 50%. Each option was paired with another, and all pairs appeared 20 times, except for the combination of the risky option and sure ¥10 option, which was presented 30 times and used to investigate the participant's risk preference. The number of trials was set as 210 in each block; thus, this experiment included 420 trials in total. We never provided the participants with information about the amounts, their probabilities, and the order of the blocks. The experiment was controlled by PsychoPy v1.80.30^25^.

### Reinforcement learning models

RL models were used to understand the learning process in relation to constructing values of the risky options. All models were designed to assign an action value to each decision-making action. Here, we consider a stimulus *i* on trial *t* for the action value $Q_{t} (i)$. The action value of a chosen action is updated based on the following equations:

1 $$Q_{t + 1} (i) = Q_{t} (i) + \alpha \,\delta_{t}$$

2 $$\delta_{t} = {\kern 1pt} r_{t} - Q_{t} (i)$$

where *α* is the learning rate that determines the speed of updating values. The outcome value $r_{t}$ is represented by 1 for a gain of ¥10, 2 for a gain of ¥20, -1 for a loss of ¥10, -2 for a loss of ¥20, or 0 for no gain or loss on trial *t*. The term $r_{t} - Q_{t} (i)$ is the prediction error (PE) described as ${\updelta }_{t}$. The initial value for each option’s action value was set as 0. The learning proceeds with a decision on each action according to the values, and the probabilities of choosing an action are calculated by the softmax function:

3 $$p_{t} (i) = \frac{{\exp (\beta \,Q_{t} (i))}}{{\sum_{i^{\prime}} \exp (\beta \,Q_{t} (i^{\prime}))}}$$

where *β* is a free parameter, inverse temperature, that represents the choice randomness.

In addition to the above standard model, we used three models that included additional parameters to test the a priori hypothesis. One model was a separate learning rate model that allowed different learning rates for a positive PE ($\delta_{t} > 0$) and a negative PE ($\delta_{t} < 0$). This model assumed that the speed of updating values could differ between the signed PEs. In this study, the learning rate for negative PE in the gain domain and the learning rate for positive PE in the loss domain are sensitive only to the value updating of the risk option, while the learning rate for positive PE in the gains and the learning rate for negative PE in the losses are used in the value updating for non-zero outcomes. If individuals have a higher learning rate for positive PE than that for negative PE, they tend to choose the risky option^9^. The subjective utility model contained a subjective utility parameter, $\kappa ,$ that could differentially weigh larger outcomes:

4 $$r_{t} = \left\{ {\begin{array}{*{20}c} 0 \\ 1 \\ { - 1} \\ {2\kappa_{G} } \\ { - 2\kappa_{L} } \\ \end{array} \;\;\begin{array}{*{20}l} {{\text{if}}\;{\text{the}}\;{\text{outcome}}\;{\text{value}}\;{\text{was}}\;{0}\;{\text{yen}}\;{\text{at}}\;{\text{trial}}\;{\text{t}}} \hfill \\ {{\text{if}}\;{\text{the}}\;{\text{outcome}}\;{\text{value}}\;{\text{was}}\;{10}\;{\text{yen}}\;{\text{at}}\;{\text{trial}}\;{\text{t}}} \hfill \\ {{\text{if}}\;{\text{the}}\;{\text{outcome}}\;{\text{value}}\;{\text{was}}\; - {10}\;{\text{yen}}\;{\text{at}}\;{\text{trial}}\;{\text{t}}} \hfill \\ {{\text{if}}\;{\text{the}}\;{\text{outcome}}\;{\text{value}}\;{\text{was}}\;{20}\;{\text{yen}}\;{\text{at}}\;{\text{trial}}\;{\text{t}}} \hfill \\ {{\text{if}}\;{\text{the}}\;{\text{outcome}}\;{\text{value}}\;{\text{was}}\; - {20}\;{\text{yen}}\;{\text{at}}\;{\text{trial}}\;{\text{t}}} \hfill \\ \end{array} } \right.$$

The subjective utility parameter is related to updating the value of the risk option and the sure ¥20 option. When the subjective utility parameter is less than 1, the risk tends to be refused in the domain of gains but accepted in the domain of losses^9^. The hybrid model included both additional parameters. Moreover, we investigated whether the RL parameters can be different in the gain and loss domains.

### Model fitting and comparison

We used a hierarchical type-II maximum likelihood estimation to fit the RL models to the data in each domain. The fitting procedure was the same as that used in a previous study^26^. With these methods, the marginal likelihood was maximized by the expectation–maximization algorithm to estimate the hyperparameters of the population-level normal distributions. The learning rate and inverse temperature parameters for each individual were transformed to sigmoidal and exponential scales, respectively. We used the Rsolnp package in R (https://cran.r-project.org/package=Rsolnp) to optimize the likelihood functions in the E-step. In the M-step, the posterior distributions were estimated by the Laplace approximation to update the hyperparameters.

The trade-off between parsimony and goodness of fit was evaluated using integrated Bayes Information Criteria (iBIC^26^). The iBIC value is twice the negative log marginal likelihood of the data with a penalty term for the number of free parameters. A model with a smaller iBIC value indicated a better prediction of the data. The marginal likelihoods were estimated via Monte Carlo sampling: for each participant, parameter values were randomly drawn from the population-level distributions and these parameter values were used to calculate likelihoods of choice data. The number of sampling was set as 1,000. These sampled likelihoods samples were averaged over the number of samples for each participant. Then, the average likelihoods were summed for all participants.

### Model recovery and parameter recovery

We performed model recovery and parameter recovery to examine whether the data generated from each learning model can be disassociated from those of other models and whether there are trade-offs between estimated parameters^21^. For each model, we made 100 simulated data with parameter sets that were sampled from the population-level normal distribution of each parameter, which was estimated simultaneously with the RL parameters by the type-II likelihood estimation. In the simulation, the values of the sampled inverse temperature parameter were increased by 1 to reduce choice randomness, which can cause poor predictability in the model^21^. The content of the simulation task was the same as that of the experimental task. We fitted each model to the simulated data using the type-II maximum likelihood estimation and computed the iBIC values. Using the iBIC values, we demonstrated that a fitting model was better in the simulated data that were generated by using a certain model.

For parameter recovery, we focused on the hybrid model that corresponds to the winning model. Using the parameter set generated by the model recovery, the correlation between true and recovered parameters and the correlation between recovered parameters were examined.

### Statistical analyses

We used several statistical tests, such as t tests, correlations, analysis of variance (ANOVA), and multiple regression. For ANOVAs, we applied the Greenhouse–Geisser correction to the degrees of freedom when the sphericity assumption was violated. In the multiple regression analysis, all the independent variables were entered into the regression equation to predict the variance of the dependent variable. In contrast, when the hierarchical regression analysis was conducted, after the average of each independent variable was subtracted from individual values of the independent variables (i.e., centering), those centering variables were entered into the linear model at Step 1. The interaction terms created by multiplying each of the centering variables were entered into the linear model at Step 2. We first checked whether the linear model with the interaction term could predict the variance of the dependent variable better than the model without the interaction term, using the F test for differences in the values of *R*^2^. Then, the slopes of the interaction terms were tested. When the slope of the interaction term was significant, the two variables were made where the values 1 SD above or below the mean of an independent variable were subtracted from individual scores of the independent variable. Then, we examined whether one independent variable was related to the dependent variable when the other independent variable was high (i.e., + 1 SD) or low (i.e., − 1 SD).

## Supplementary Information

Supplementary Information.

## Data Availability

The raw data supporting the conclusions of this manuscript will be made available by the corresponding author on reasonable request.
