# Supplementary material for: A learning mechanism shaping risk preferences and a preliminary test of its relationship with psychopathic traits
Source: Sci Rep. 2021 Oct 21;11:20853. doi: 10.1038/s41598-021-00358-8 (PMC8531311; doi:10.1038/s41598-021-00358-8)
Supplement: Supplementary file 1 — Supplementary Information. [file 41598_2021_358_MOESM1_ESM.pdf]

Supplementary information

Title: A learning mechanism shaping risk preferences and a preliminary test of its relationship with psychopathic traits.

Author names: Takeyuki Oba, Kentaro Katahira and Hideki Ohira

Corresponding Author: Takeyuki Oba
E-mail: takeyuki.oba@gmail.com

This PDF file includes the following:
Supplementary Figure S1 and Figure S2
Supplementary Tables S1

Supplementary figures

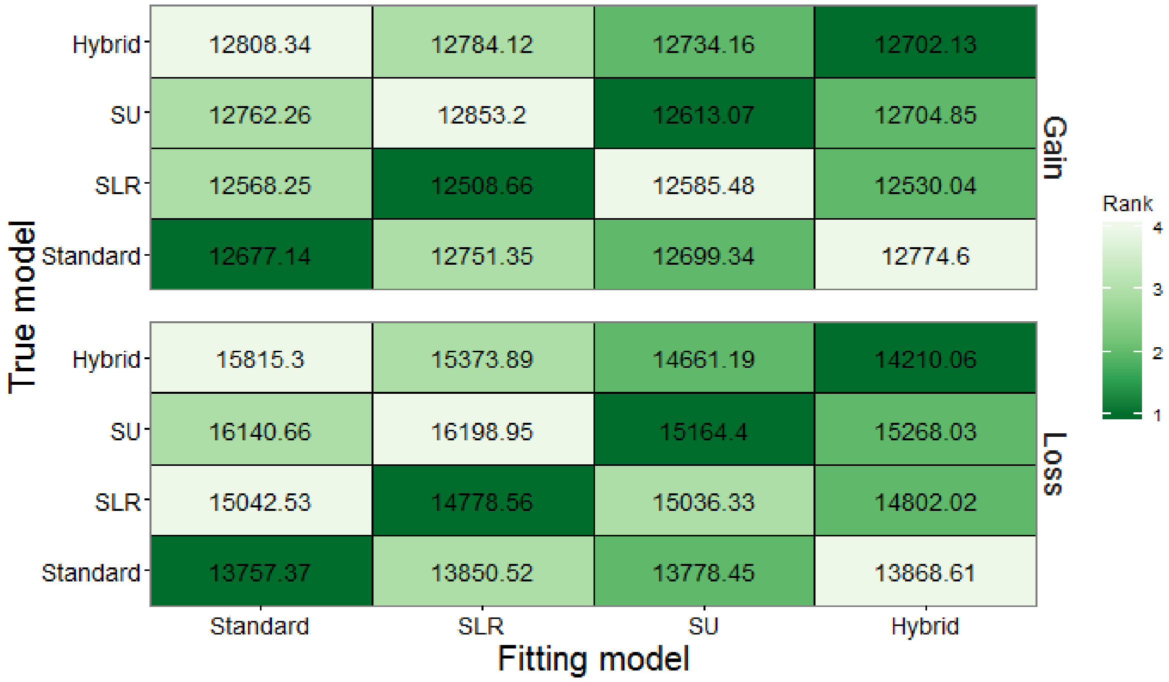

Supplementary Figure S1. Confusion matrix of model recovery. Each panel shows the iBIC value. The green gradient indicates the ranking of the iBIC values in each row. SLR = Separate learning rate model, SU = Subjective utility model.

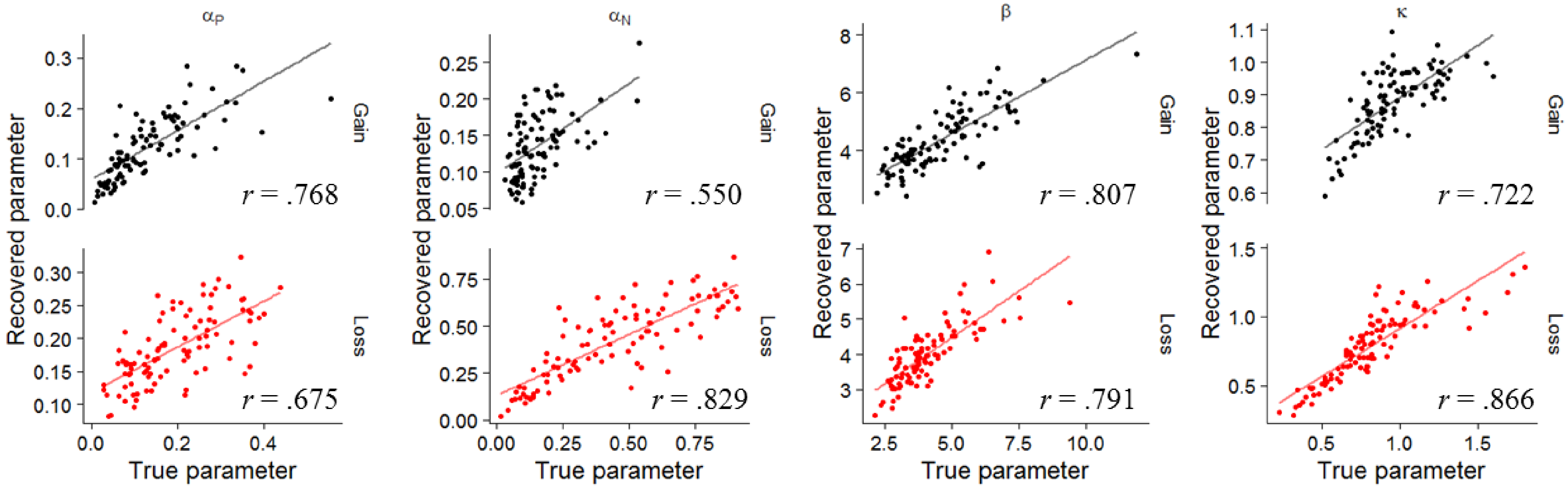

Supplementary Figure S2. Correlations between the recovery parameters and the true parameters for the hybrid model. The left side of the figure shows the correlations in the learning rates, while the right side of the figure shows the correlations in the inverse temperature and the subjective utility parameter. The upper panels represent the results in the gain domain, and the bottom panels represent the results in the loss domain.

Supplementary table

Supplementary Table S1. The correlations between recovered parameters for each domain

	Gain					Loss			
	α_{GP}	α_{GN}	β_G	κ_G		α_{LP}	α_{LN}	β_L	κ_L
α_{GP}		-.131	-.243*	.019	α_{LP}		-.117	.136	.248*
α_{GN}			-.049	.184	α_{LN}			-.090	-.003
β_G				.083	β_L				-.123
κ_G					κ_L				

The subscripts represent the following: P = positive PE, N = negative PE, G = gain domain, L = loss domain. *$p < .05$.
